# Complete mitochondrial genome of the Ijima's Sea Snake (*Emydocephalus ijimae*) (Squamata, Elapidae)

**DOI:** 10.1080/23802359.2019.1641438

**Published:** 2019-07-22

**Authors:** Chang-Ho Yi, Jaejin Park, Takahide Sasai, Hye Seon Kim, Jong-Gwan Kim, Min-Seop Kim, In-Young Cho, Il-Hun Kim

**Affiliations:** a National Marine Biodiversity Institute of Korea, Seochun, Republic of Korea;; b Seoul National University, Seoul, Republic of Korea;; c Kangwon National University, Chuncheon, Republic of Korea;; d University of the Ryukyus, Nishihara, Japan

**Keywords:** *Emydocephalus ijimae*, mitochondria genome, complete, elapid snakes, Hydrophiinae

## Abstract

In this study, we provide the first report of the complete mitochondrial genome of *Emydocephalus ijimae*. The mitogenome length is 18,259 bp and includes 13 protein-coding genes, two rRNA genes, 22 tRNA genes, and three non-coding regions. The sequence presented could be very useful for further phylogenetic and evolutionary study.

Sea snakes, a representative marine reptile group in the family Elapidae, comprise 63 Hydrophiinae species (true sea snakes) and eight Laticaudinae species (sea kraits) (Reptile Database 2019). Although the complete mitochondrial genome of three sea kraits is known (Kim et al. 2018), there are no published studies for any species of the subfamily Hydrophiinae.

To investigate the phylogenetic relationships of the three subfamilies of Elapidae snakes, we determined the complete mitochondrial genome of *Emydocephalus ijimae* (Stejneger 1898). Although genus *Emydocephalus* is not clearly distinguished morphologically (Rasmussen and Ineich 2010; Lukoschek & Sanders 2010), genetic analysis results for additional analysis are extremely rare. The specimen of *E. ijimae* was collected from the port in Serakaki, Onna, Kunigami District, Okinawa, Japan (26°30′18.24″ N and 127°52′48.37″ E) on August 05, 2013. The specimen was preserved in 70% ethanol and kept at the National Marine Biodiversity Institute of Korea (Voucher No. MABIK AR00000052).

We extracted the whole genomic DNA from the muscle tissue of the specimen using the phenol-chloroform isoamyl alcohol method as described by Asahida et al. (1996) and proteinase K (Bioneer, Daejeon, South Korea). We designed sixteen primer sets to amplify the segments by long range PCR. The whole mitochondrial DNA was sequenced using the Sanger method using a 3730xl DNA analyzer (The Applied Biosystems, CA, USA). Sequencing was performed at Macrogen Inc. (Seoul, South Korea). To assemble and annotate the mitochondrial DNA sequence, we used Geneious 9.0.4 (Biomatters Ltd., Auckland, New Zealand), tRNA Scan-SE1.21 (http://lowelab.ucsc.edu/tRNA Scan-SE/) (Lowe and Eddy 1997), and Dual Organellar GenoMe Annotator (DOGMA) (Wyman et al. 2004). The phylogenetic tree for E. ijimae and other related elapid snakes was constructed with PAUP* v4.0b10 (Swofford 2003) using the 13 protein-coding genes obtained in this study and from GenBank (Figure 1). We applied the Bayesian inference and maximum likelihood method to construct the tree with 1,000 bootstrap replicates.

**Figure 1.**
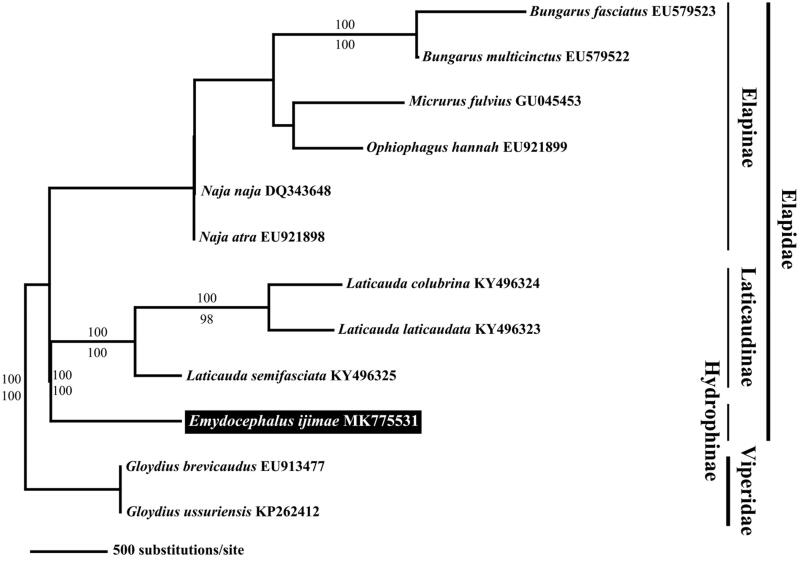
Maximum-likelihood (ML) tree based on the 13 mitochondrial protein-coding genes of *Emydocephalus ijimae* with other nine elapid snakes. We used each two species in Viperidae as the outgroup. The accession number of the mitogenomes, which obtained from GenBank, indicated after the scientific name of each species. On each branch, Bayesian posterior probabilities (above) and bootstrap value (below) are denoted.

The total length of the *E. ijimae* mitochondrial genome was 18,259 bp (GenBank Accession No. MK775531), with a base composition of 33.1% A, 27.6% T, 12.6% G, and 26.8% C, which showed an A-T rich (60.7%) feature. The mitogenome comprised 13 protein-coding genes, 2 rRNA genes (12S and 16S rRNA), 22 tRNA genes, and 3 non-coding regions of two control regions and an L-strand replication origin. The arrangement pattern and transcribing directions of the mitogenome were identical to those of other elapid snakes (three laticaudini species and *Naja Naja*), which were previously reported (Yan et al. 2008; Kim et al. 2018).

The phylogenetic tree for *E. ijimae* and other related elapid snakes shows that true sea snakes and other elapid snakes including sea kraits (Genus *Laticauda*) form a sister group (Figure 1). Additionally, this result is similar to the previous result by Slowinski and Keogh (2000) regarding the phylogenetic relationships of elapid snakes based on the cytochrome b sequence and that of Keogh (1998) regarding the phylogeny of elapid snakes based on 16S rRNA and cytochrome b.
